# Extracellular Vesicles and Their Relationship with the Heart–Kidney Axis, Uremia and Peritoneal Dialysis

**DOI:** 10.3390/toxins13110778

**Published:** 2021-11-04

**Authors:** Carolina Amaral Bueno Azevedo, Regiane Stafim da Cunha, Carolina Victoria Cruz Junho, Jessica Verônica da Silva, Andréa N. Moreno-Amaral, Thyago Proença de Moraes, Marcela Sorelli Carneiro-Ramos, Andréa Emilia Marques Stinghen

**Affiliations:** 1 Experimental Nephrology Laboratory, Basic Pathology Department, Universidade Federal do Paraná, Curitiba 81531-980, Brazil; carolina.amaral1@ufpr.br (C.A.B.A.); regidacunha@gmail.com (R.S.d.C.); 2 Laboratory of Cardiovascular Immunology, Center of Natural and Human Sciences (CCNH), Federal University of ABC, Santo André 09210-580, Brazil; carolina.junho@gmail.com (C.V.C.J.); jessica.veronica12@hotmail.com (J.V.d.S.); msorelli@gmail.com (M.S.C.-R.); 3 Graduate Program in Health Sciences, School of Medicine, Pontifical Catholic University of Paraná, Curitiba 80215-901, Brazil; andrea.moreno@pucpr.br (A.N.M.-A.); thyago.moraes@pucpr.br (T.P.d.M.)

**Keywords:** extracellular vesicles, cardiorenal syndrome, peritoneal dialysis

## Abstract

Cardiorenal syndrome (CRS) is described as primary dysfunction in the heart culminating in renal injury or vice versa. CRS can be classified into five groups, and uremic toxin (UT) accumulation is observed in all types of CRS. Protein-bound uremic toxin (PBUT) accumulation is responsible for permanent damage to the renal tissue, and mainly occurs in CRS types 3 and 4, thus compromising renal function directly leading to a reduction in the glomerular filtration rate (GFR) and/or subsequent proteinuria. With this decrease in GFR, patients may need renal replacement therapy (RRT), such as peritoneal dialysis (PD). PD is a high-quality and home-based dialysis therapy for patients with end-stage renal disease (ESRD) and is based on the semi-permeable characteristics of the peritoneum. These patients are exposed to factors which may cause several modifications on the peritoneal membrane. The presence of UT may harm the peritoneum membrane, which in turn can lead to the formation of extracellular vesicles (EVs). EVs are released by almost all cell types and contain lipids, nucleic acids, metabolites, membrane proteins, and cytosolic components from their cell origin. Our research group previously demonstrated that the EVs can be related to endothelial dysfunction and are formed when UTs are in contact with the endothelial monolayer. In this scenario, this review explores the mechanisms of EV formation in CRS, uremia, the peritoneum, and as potential biomarkers in peritoneal dialysis.

## 1. Introduction

Extracellular vesicles (EVs) have been widely investigated for their role in intercellular communication and as potential biomarkers, particularly in inflammatory pathological conditions. Cardiorenal syndrome results from interrelated heart and kidney injuries, which leads to an accumulation of uremic toxins in the body, especially with the progression of chronic kidney disease (CKD) [1,2,3]. However, the participation of EVs in CRS has not been fully elucidated. Clinical and in vitro studies have shown that uremic toxins induce the formation of EVs [4,5,6,7,8]. In this review, we address the role of EVs in CRS, especially their relationship with uremic toxins and kidney dysfunction. In addition, EVs from the peritoneal membrane in ESRD patients undergoing peritoneal dialysis are potential biomarkers. We also discuss the classification of EVs and the main methods for isolating and characterizing EVs, including electron microscopy, proteomics, lipidomics, transcriptome and metabolomics analyses, Fourier transform infrared (FTIR), and Raman spectroscopies, as well as possible use of EVs as biomarkers of cell injury and the therapeutic strategies to avoid their formation.

## 2. Cardiorenal Syndrome: Role of Uremic Toxins (UTs) in Peritoneal Dialysis

CRS is a set of diseases with clinical and metabolic consequences triggered by acute and/or chronic heart failure (CRS I and II) or acute and/or chronic kidney disease (CRS III and IV) resulting in injury to the other organs (Table 1). Failure of both organs can also occur simultaneously as a consequence of a systemic disease (CRS V). Despite the current categorization of the CRS into these five groups, substantial overlap is observed between the different types [9].

Type 1 CRS is defined as acute cardiorenal syndrome, in which an acute cardiac insult contributes to developing an acute kidney injury (AKI). Acute decompensated heart failure (ADHF) is the most common cause of type I CRS with hemodynamic mechanisms playing a major role in causing AKI [1]. Nevertheless, non-hemodynamic mechanisms have been proposed as contributors to type I CRS, namely chronic inflammation and overproduction of reactive oxygen species [11,12]. In addition, sodium uptake and water retention occurs as a compensatory mechanism, but also contributes to renal congestion [1]. Its treatment is based on drugs to restore the cardiac function, alleviate congestion and normalize the excess reabsorbed sodium [13]. Kidney function in CRS type 1 is normally restored when normal hemodynamics are recovered [14].

Type 2 CRS is characterized by chronic heart failure causing chronic kidney disease (CKD). The underlying mechanisms involve chronic kidney hypoperfusion, increased renal vascular resistance, overactivation of the SNS and RAAS, increased venous pressure, volume overload, endothelial dysfunction and inflammation [12]. This subtype of CRS is very common and has been described in up to 63% of patients with CRS in some reports [15]. The most common mechanisms believed to be involved in the development of type 2 CRS are neurohormonal activation, renal hypoperfusion, venous congestion, inflammation and oxidative stress [12].

Type 3 CRS is defined when an AKI event causes or contributes to developing acute heart injury, such as an event involving renal ischemia and reperfusion injury (IRI) [16]. Ischemia and reperfusion injury (IRI) commonly follow the acute renal event. However, the pathophysiology of the type CRS 3 is also complex and not completely understood, but it is known for certain that it is multifactorial and associated with inflammatory processes, oxidative stress, neurohormone secretion, hyperactivity of the SNS and RAAS, volume overload, metabolic acidosis and electrolyte disturbances [12]. Similar to CRS type 1, type 3 has adaptive hemodynamic mechanisms to circumvent the systemic injury which can also cause sodium retention and volume overload which contributes to heart disease [14].

Type 4 CRS is defined as a chronic reno-cardiac disease characterized by cardiovascular impairment in CKD patients. CKD contributes to many cardiovascular diseases (CVD), being considered one of the most important risk factors [17]. There are a multitude of interconnected factors within the mechanisms behind type 4 CRS. They include an essential role of inflammation and oxidative stress, as well as the effects caused by the presence of uremic toxins (UT) [18]. Other well described factors involved in the process are: hyperphosphatemia, high parathyroid hormone (PTH) serum levels, vitamin D deficiency, elevated fibroblast growth factor 23 (FGF23) plasma levels, in addition to the presence of adenosylhomocysteine, hypervolemia, and anemia, among others. As CKD progresses, this accumulation of phosphate increases FGF23, which is a direct mechanism in promoting hypertrophy, remodeling, and contractile alterations in the heart [19,20,21,22,23,24].

Finally, type 5 CRS occurs as a consequence of a systemic disease as cardiac and renal injuries occur simultaneously. Sepsis, diabetes, hepato-renal syndrome, immune disorders, cancer, and more recently COVID-19, are examples of these conditions which can lead to type 5 CRS [14,25]. Many of these alterations happen as an immediate response to systemic damage stimuli which the body tries to combat [26]. Given the heterogeneity in the etiology of the underlying systemic diseases associated to type 5 CRS, the mechanisms involved in this CRS subtype are complex to summarize. The role of UT accumulation needs to be elucidated in type 5 CRS, since it can be involved as a cause or can be caused by CKD [12].

UTs are compounds that accumulate in CKD plasma patients, leading to different injuries in the organs compromising several biological functions, such as renal and cardiovascular function [27,28,29]. These compounds are divided into three main groups: water-soluble, small and medium sized molecules, and protein-bound uremic toxins (PBUT). Water-soluble (<500 Da) and medium (>500 Da) components do not bind to proteins [30]; while PBUTs are low molecular weight molecules which utilize proteins such as albumin for transportation, and as a result of their size cannot be removed by the dialysis [2].

UT accumulation can be observed in all types of CRS [3,12,31,32]. Following renal injury, the structural damage in the kidney compromises renal function resulting in a reduction in GFR and/or subsequent increased proteinuria [2], this can cause an increase in UT accumulation in the blood further compounding the functional and structural deterioration of the kidneys and other organs [3]. Although it has been pointed out that this accumulation of toxins causes a primary injury to the kidney, some studies have suggest (i.e., Di Lullo et al.) that it could be considered a type 5 CRS since the uremic compounds can also directly cause damage to the cardiac tissue, featuring a systemic disease [3,12].

In addition, some studies have pointed out the role of creatinine, uric acid, inorganic phosphate, and FGF23 regarding water-soluble and medium compounds and CRS. Creatinine is a renal disease biomarker and uric acid accumulation is also associated with the development of atrial fibrillation, heart failure, and hypertension, as well as RAAS activation [31,32]. These compounds (creatinine and uric acid) are already being used as biomarkers of CRS in the medical field, and their association with RAAS blockers is quite important. Inorganic phosphate (Pi) accumulation is also connected to a high incidence of CVD mortality observed in CKD patients [33,34]. This occurs as dialysis patients show positive correlations of serum Pi x vascular calcification [35]. This calcification effect is observed as some osteogenic genes are increased in smooth muscle cells after Pi stimulation [36]. In addition to calcification, Pi also increases the risk of heart failure and systolic dysfunction [33]. Neves et al. demonstrated that myocardial hypertrophy is followed by hyperphosphatemia in a model of renal failure [37].

The accumulation of medium-sized UT also contributes to renal structural damage, reducing the GFR and cardiac function [18]. Patients with advanced CKD present with low GFR and 1000-fold elevated FGF23 levels [38]. This has adverse effects on the heart via an independent mechanism, promoting cardiac hypertrophy and contractile dysfunction [39,40]. The Pi and FGF23 levels appear to be inter-related by their mechanisms. The Pi levels increase as soon as the renal function decreases, and plasma FGF23 concentration increases due to significant changes in phosphate or serum PTH concentration. One study considered FGF23 a secondary UT, since it only increases after phosphate accumulation [41]. There are relevant findings regarding Pi and medium-sized UT inducing hypertrophy of the myocardium, hyperplasia of cardiomyocytes, and interstitial fibrosis and vessels [2].

The most damaging UTs in the blood of CKD patients are bound to serum albumin (PBUT), evidenced by impaired dialytic function in these patients. Due to its difficult removal by the dialysis membrane, these compounds can cause structural damage in heart and kidney tissues as they accumulate. Their damage is mainly associated with the loss of renal function and CKD progression [27,42]. Increased circulating levels of the PBUT indoxyl sulfate (IS) and p-cresyl sulfate (PCS) are observed in patients with CKD. In vitro, it was demonstrated that IS enhanced pro-inflammatory cytokine gene expression, such as TNF-α, IL-6, and IL-1β, in a monocytic cell line [43]. Glomerular injury induced by IS administration was studied to examine the interaction of podocyte injury. After treatment with IS, Iichi et al. found that mouse podocytes exhibited a pro-inflammatory phenotype, perturbed actin cytoskeleton, decreased expression of podocyte-specific genes, and decreased cell viability, contributing to progressive glomerular injury [44] and to the advancement of CRS type 4, for example. PBUT generally lead to arrhythmias, fibrosis, hypertrophy and reduced anti-oxidative protein expression in cardiomyocytes [43,45,46]. The main examples of PBUTs are PCS and IS. The concentration of PCS in the blood of healthy individuals is low but is increased in patients with ESKD. PCS accumulation has been associated with compromised vascular injury, mortality, and activation of leukocyte free radical production [47]. Increased levels of IS have been related to the progression of CKD and development of CVD [48,49], along with the increased expression of adhesion molecules in endothelial cells and oxidative stress that eventually leads to endothelial damage [50,51,52]. Besides, Nakano et al. (2019) suggests that IS is able to induce immune dysfunction through activation of pro-inflammatory macrophages [53]. In addition, Kim et al. (2019) demonstrated the interaction between the aryl hydrocarbon receptor (AhR), nuclear factor kappa-light-chain enhancer of activated B cells (NF-κB) and the suppressor cytokine signaling (SOCS) 2, which is important for the production of TNF-α in human macrophages stimulated by IS [54].

According to the World Health Organization (WHO), CVD is the main cause of death worldwide [55]. Almost 45% of these deaths have been observed in renal failure patients. It is undeniable that the CRS studies have attracted curiosity and investment by the pharmaceutical industry which increasingly seeks to maintain the quality of life of CVD patients. Investment in new technologies has received much praise and increased interest in recent years. Understanding the heart–kidney axis is fundamental to continue discoveries in the medical field. Studying uremic toxins’ cellular and molecular effects may elucidate new biomarkers and therapeutic targets in CKD.

Due to UT accumulation and CKD progression, end-stage patients require renal replacement therapy (RRT) which includes renal transplantation, hemodialysis (HD), or peritoneal dialysis (PD). HD and PD work by removing solutes, toxins, and water, restoring electrolyte balance, and correcting acidosis. However, while HD is based on blood passing through an extracorporeal circuit through vascular access, PD involves an exchange of solutes and water between blood from the peritoneal capillaries and a solution instilled into the peritoneal cavity through a catheter using the peritoneal membrane as the dialysis surface [56].

**Figure 1:**
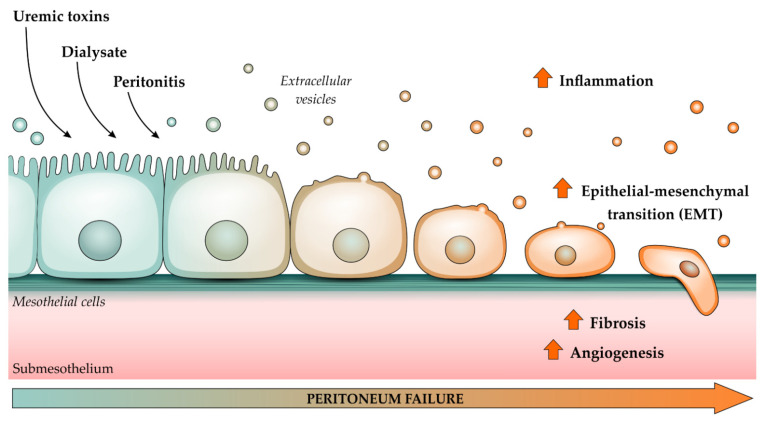
Effects of uremic toxins (UT), dialysate, and peritonitis on mesothelial cells. Mesothelial cells show a progressive loss of the epithelial phenotype as soon as peritoneal dialysis (PD) starts, and acquire myofibroblastic characteristics through an epithelial–mesenchymal transition (EMT) and gain a high migratory and invasive capacity, contributing to fibrosis, angiogenesis and subsequent peritoneum failure. Stressed or activated mesothelial cells can shed extracellular vesicles which can be potentially used as biomarkers of damage and even dialysis efficiency.

PD is a high-quality and home-based dialysis therapy for patients with end-stage renal disease (ESRD) and is based on the semi-permeable characteristics of the peritoneum [57]. PD has the advantage of ensuring greater freedom and independence to the patient [56]. As a continuous therapy, PD constantly removes solutes and water, allowing for a less restricted diet compared to their counterparts on hemodialysis, and provides great preservation of residual renal function, which is critical to the well-being and survival of the patients [58]. However, long-term exposition to bioincompatible PD solutions, along with the uremic state common to ESRD, results in dysfunction of the peritoneum, characterized by chronic inflammation, fibrosis, and loss of dialysis and ultrafiltration capacity. Uremic patients have an increase in the average thickness of the peritoneum and altered expression of proteins even before PD treatment, including transforming growth factor (TGF)-β and vascular endothelial growth factor (VEGF), which is involved in structural changes in the kidneys and peritoneum [59,60,61]. This suggests that uremic toxins themselves impact the peritoneal membrane, and their effects are exacerbated by the chronic exposure to bioimcompatible PD fluids along with recurrent cases of PD related infections. It has additionally been shown that mesothelial cells show a progressive loss of the epithelial phenotype as soon as PD starts and acquire myofibroblastic characteristics through an epithelial–mesenchymal transition (EMT) [62]. Mesothelial cells that go through the EMT process acquire a high migratory and invasive capacity, allowing them to invade submesothelial stromal cells, which in turn contribute to peritoneal fibrosis, angiogenesis and eventually technique failure (Figure 1) [63].

The functional capacity of the peritoneal membrane is evaluated through the peritoneal equilibration test (PET). PET was developed by Twardowski [64] and characterizes the transport rates of the patient’s peritoneum, enabling prescription adaptations for dialysis treatment based on the characteristics of each patient as they eventually show signs of damage to the peritoneum [57,65]. Changes in the PET reveal membrane deterioration with consequent failure in ultrafiltration, but the information displayed by the test is inadequate and often late [65]. Morphological alterations can also be evaluated through peritoneal biopsies, but this procedure is cumbersome, invasive, may not be representative of the entire peritoneal membrane, and is consequently rarely performed for diagnosis [59]. Thus, it is necessary to search and standardize other methods for diagnosing peritoneal membrane dysfunction.

Peritoneal tissue in contact with hypertonic dialysis solutions and uremic toxins secrete extracellular substances and vesicles which can be analyzed from the dialysate. These substances and vesicles are potential biomarkers of PM integrity [66].

## 3. The Importance of Extracellular Vesicles in Heart/Kidney Axis and Peritoneal Dialysis

**Figure 2:**
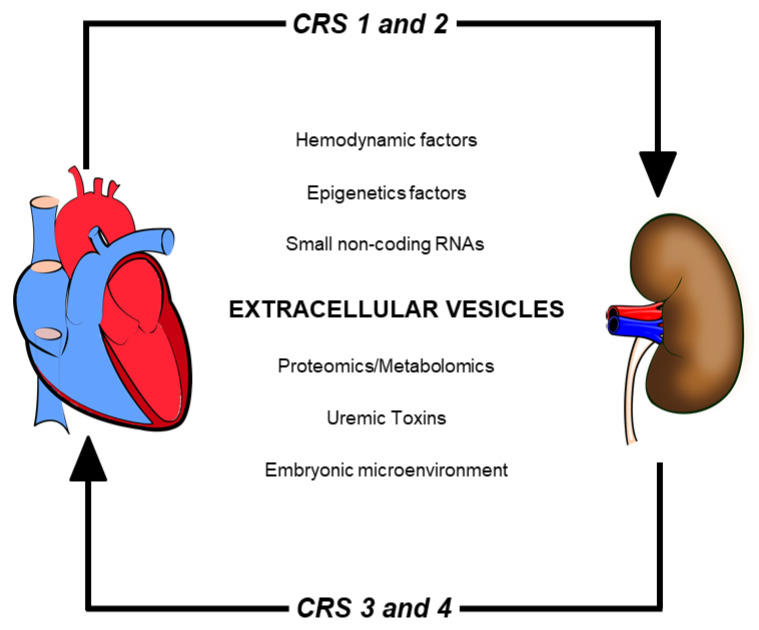
Multi-factorial mechanisms implicated in the pathogenesis of cardiorenal syndromes. Some factors modulate the heart–kidney axis, including hemodynamic parameters, uremic toxins, gene reprogramming, and extracellular vesicles.

Robert Bright discovered communication between the heart and kidneys in 1836 when he observed cardiac events in patients with renal disease and albuminuria [67], thereafter many mechanisms have been shown to participate in this axis. The main mechanisms are related to hemodynamic homeostasis, such as activation of RAAS and SNS, as mentioned previously. One crucial mechanism recently studied is the contribution of extracellular vesicles (EVs). They are known as facilitators of communication in diverse cellular processes [68] (Figure 2).

The EVs are vesicular nano-sized membrane-enclosed structures composed of a lipid bilayer (such as the cell plasmatic membrane) which transport body cargo such as DNA, RNA, and proteins from their cell of origin and have the ability to physiologically and pathologically influence their cell of origin and other cells [68,69,70]. They can be detected in plasma, urine, and other body fluids of healthy people [71], and their levels are increased in various diseases, mainly reflecting the injury suffered in these tissues [72]. As their composition depends on the pathophysiological and functional state of their cell of origin, they have been studied as potential biomarkers in several diseases, especially cardiovascular [73,74,75], immune [76,77,78,79], cancer [80,81,82,83,84], viral infection, including COVID-19 [85,86], CKD [4,87,88,89,90], and recently in peritoneal dialysis [57,65,66,91,92].

According to the International Society of Extracellular Vesicles (ISEV), EVs are all vesicles released from a cell which can be classified based on their mechanism of formation, mode of release from the cells and size [93,94]. Studies have broadly divided EVs into three main groups: microvesicles (MVs) (also called microparticles), exosomes, and apoptotic bodies.

MVs are 100–1000 nm sized vesicles generated from plasma membrane remodeling induced by activation, stress, or cell death [95,96]. MVs contain cargo from the cytoplasm such as proteins and nucleic acids (mRNA, miRNA, and other non-coding RNAs) with membrane-specific antigens [97]. Thus, MVs are considered ideal biomarkers of cell and tissue damage because of these properties and because they retain their cell of origin characteristics [71,98,99]. Therefore, an assessment of MVs in biological samples can be used to identify early tissue damage [72]. Many studies have also demonstrated the ability of MVs to serve as intercellular signalers and capable of inducing pro-inflammatory, pro-apoptotic, and pro-fibrotic responses in target cells [88,100,101,102]

Exosomes are vesicles sized 40–120 nm which are formed from the fusion of multivesicular bodies and plasma membranes in a process called “reverse endocytosis” characterized by Johnstone et al. in 1987 [103]. The biogenesis of exosomes is controlled by several factors, including activating specific receptors and signaling pathways [94]. Exosomes contain functional microRNAs (miRNAs) and small RNAs which can transfer between circulating cells. Exosomes can interact with recipient cells through endocytic uptake, a direct fusion of the vesicles to the cell membrane, or by adhesion to the cell surface—mediated by the interaction of a lipid–ligand receptor when they are released into the extracellular environment [104,105]. The apoptotic bodies are at least 50–2000 nm sized vesicles released by dying cells. As the cells die, they generate many membrane-bound vesicles with organelles or nuclear content [70].

**Figure 3:**
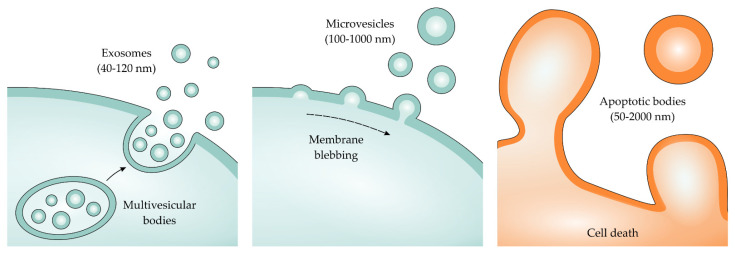
Representation of exosomes, microvesicles, apoptotic bodies, and their mode of release from cells.

The presence of exosomes has particularly been described in many body fluids such as plasma, urine, semen, breast milk, and amniotic fluids; and is evolutionarily conserved in several groups of eukaryotes (especially mammals) [106]. Exosomes in renal tissue are mostly released by the epithelium or podocytes, transmitting acute and CKD messages, and structural and molecular biomarkers (Figure 3) [107,108]. It is also unknown if nephron loss in CKD results in the formation of urine EVs (uEVs). These exosomes are sensitive to renal damage and can be mainly released by the thick ascending limb of the loop of Henle (salt and water downstream nephron segments). uEVs from this nephron segment may be more likely to be entrapped, once they are collected by the collecting duct appearing in urine [109].

In kidney diseases, proximal tubular cell proteins have been found in vesicles from the collecting ducts, clarifying the local transmission connection [68]. Aquaporin-2 and Fetuin-A found in exosomes obtained from the collecting duct have a physiological role reflected in cellular expression in the kidney [110,111]. Fetuin-A was increased in uEVs in a cisplatin-induced AKI rat model [110]. Fetuin-A was detectable after 2 days of increasing creatinine levels. After establishing the biomarker, it was tested in an ischemia and reperfusion (IR) model and AKI patients [110]. The water channel aquaporin-1 was found to decrease in uEVs in animals after renal IR and in patients undergoing kidney transplantation, being detectable from 6 h after IR [112].

The presence of some proteins (i.e., histone-lysine N-methyltransferase (MLL3), α-microglobulin/bikunin precursor (AMBP), and voltage-dependent anion-selective channel protein 1 (VDAC1)) in uEVs were observed in samples from diabetic nephropathy patients [113]. We were able to find several changes concerning EVs during AKI. Alvarez et al. observed the presence of NGAL (an AKI biomarker) in urinary exosomes from patient samples, also reinforcing the high sensitivity of the uEVs appearing in renal disease [114]. Jeon et al. observed the contribution of the miRNAs in EVs derived from injured podocytes on tubular apoptosis and dysfunction using HK2 cells, identifying miRNA-424 and -149 as having a role in this process [115].

The role of the UTs in the release of EVs, have mostly been studied in the setting of endothelial dysfunction leading to a release of endothelial-derived microvesicles (EMVs). In vitro and clinical studies observed that PCS was able to induce spreading of EMVs in cell culture as well as increase the levels of EMVs in hemodialysis patients [116]. EMVs also have an important role in patients with ESRD, mainly modulating vasorelaxation, and decreasing endothelial nitric oxide (eNO) release [99]. Favretto et al. observed the formation of different-sized EVs from endothelial cells generated by PCS, IS, and Pi treatments in vitro. In addition, it stimulated cell adhesion markers in PCS and Pi-induced EVs and VCAM-1 expression in PCS and IS-induced EVs [4].

When exposed to an increased hemodynamic load due to physiological stress such as CRS, the heart responds adapting to new operating conditions; however, prior to this adaptation, it responds with a particular communication using EVs to mediate the various cell populations [117,118]. This interaction was previously described by Waldenström et al. when internalization of cardiomyocyte exosomes was observed in fibroblasts and endothelial cells [119]. This study showed the presence of genetic material of cardiomyocytes inside the cytoplasm of other cells types, promoting modification of gene expression. In this context, the EV interaction between cardiomyocytes and fibroblasts was important in the progression of chronic heart failure and is given by the transport of the miR-217 from cardiomyocytes to fibroblasts promoting its proliferation and consequent fibrosis [120]. Cardiomyocytes have also been show to release exosomes internalized by endothelial cells containing miRNAs in order to increase angiogenesis after stress (miR-17,19a,19b,20a,30c,126) [121].

Fibroblasts are also involved in cell to cell exosome communication in the heart. Cosme et al. demonstrated that cardiomyocyte viability was increased if fibroblast exosomes were added to healthy cardiomyocytes before hypoxia, otherwise it was reduced [122]. This suggests the contribution of fibroblast exosomes. Fibroblast EVs also transport significant miRNAs to cardiomyocytes, such as miR-21 involved in cardiomyocyte hypertrophy [123]. In addition, the role of fibroblast exosomes containing miRNA-27a, miRNA-28a, and miRNA-34a are also involved in cardiomyocyte hypertrophy, promoting the expression of hypertrophic markers (ANP and β-MHC) after MI [124].

Cardiac exosomes can release important signaling components during stress. Angiotensin II type 1 receptors (AT1R) are transferred by the myocardium into the exosome after pressure overload in mice [74]. Functional EVs carrying these receptors were found in the circulation of these animals, providing evidence that this trafficking occurs during stress. In addition, coronary serum exosomes from patients with MI demonstrated lower miR-939-5p levels when compared to control patients [125]. This miRNA expressed in cardiomyocytes exosomes is responsible for endothelial angiogenesis through nitric oxide (NO) pathways.

Limited studies have demonstrated the role of EVs in CRS. Levin-Schwartz et al. showed the interactions between urinary exosomal miRNAs (exo-miRs) as emerging biomarkers of renal health and cardiorenal outcomes in early childhood, in which a relationship between blood pressure, GFR, sodium/potassium levels, and 20 exo-miRs was found [126].

Santelli et al. also observed cardiorenal connection with EVs. As hypertension and renovascular hypertension lead to renal injury, these authors studied EVs from urine and plasma of patients, and analyzed specific markers including p16 (renal senescence marker), calyxin (from podocytes), and others. They identified a high quantity of p16-EVs in the urine of hypertensive patients [127]. In addition, some podocyte-specific proteins such as nephrin, podocin, cytokeratin 8, claudin-1, pax-2, and UCH-L1 reflected in podocyte-uEVs were associated with an increase in nephrinuria during preeclampsia [128]. Furthermore, the levels of nephrinuria were positively correlated with proteinuria, indications of podocyte injury reflecting secondary kidney damage, and potential involvement in CRS types 1 and 2. [128]. Erdbrügger et al. consider endothelial microparticles (EMPs) of renal origin a predictor of cardiovascular mortality from studying 81 dialysis patients and observing cardiovascular parameters [98,99].

Endothelial damage is already marked in the early stages of CKD, directly leading to cardiovascular risk from atherosclerosis and arterial stiffness [17]. Dursun et al. showed that CD144-EMP was positively correlated to blood pressure and PTH concentration in CKD patients and negatively correlated to GFR and albumin, which suggests endothelial dysfunction in these patients [129]. Amabile et al. showed a close correlation between EMPs and vascular dysfunction in vivo, suggesting a role of EMPs in disease progression and as potential risk factor in the occurrence of cardiovascular events in patients with ESRD [89]. A pilot study by the same group in 2012 demonstrated that EMP plasma levels in patients with ESRD are an independent predictor of all-cause and CV mortality [98]. Buendía et al. showed that damage to endothelial cells results in EMPs with a high content of calcium and bone morphogenetic protein-2 capable of inducing calcification and osteogenic differentiation in vascular smooth muscle cells [88].

**Figure 4:**
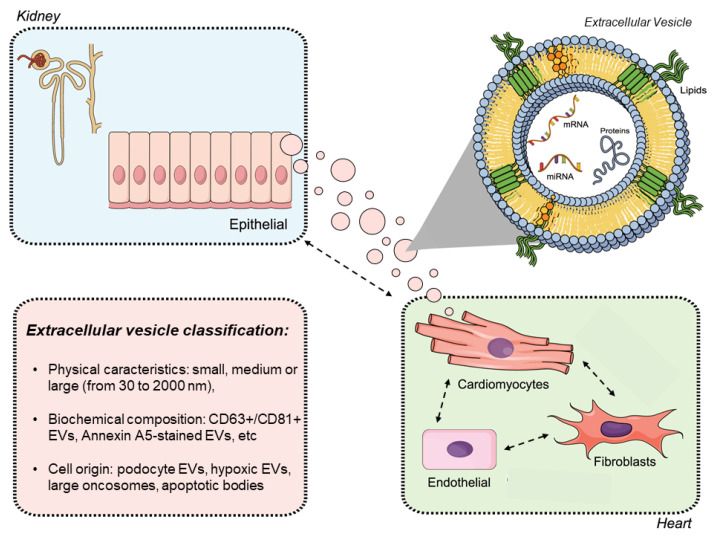
Interaction of extracellular vesicles in the heart–kidney axis. Extracellular vesicles may play an essential role in the cross-talk between the heart and kidneys. The role of extracellular vesicles (EVs) takes on special significance in the context of cardiorenal syndrome (CRS) as it promotes bidirectional crosstalk. The EVs could be released from the kidney and target the heart. It may be possible to observe a connection between organs and an intra-organ connection at the same time. The EVs are presented in different sizes and biochemical compositions, depending on the molecules delivered to target organs.

The contribution of EVs in the kidney–heart axis is largely unexplored. Thus, understanding the role of EVs in the heart–kidney connection should not only focus on their role as a biomarker, but also as a therapeutic target (Figure 4). Unfortunately, the isolation of EVs in clinical practice is still in its infancy, making more specific studies and immediate approaches difficult. The advancement and management of CRS are challenging due to the diversity and complexity of the pathophysiological interactions between these two organs. Further studies related to EVs and genetic mechanisms are needed to improve clinical outcomes in patients with cardiac and renal diseases.

Recent studies have begun to explore the use of EVs in PD (Table 2); however, more studies with a higher number of patients are needed. Using EVs obtained from patient PD fluid exchange provides an easy and convenient non-invasive method to assess biomarkers and evaluate early changes in the peritoneal membrane function.

Carreras-Planella et al. isolated EVs from the peritoneal dialysate through size exclusion chromatography and performed a protein analysis by mass spectrometry. Interestingly, the protein profile of these vesicles showed notable differences between patients who had started dialysis recently and patients who had been undergoing treatment for a longer time, demonstrating that in addition to the vesicles per se, proteomic analysis of these vesicles can be an important tool for identifying the extent of damage and ultrafiltration failure [57].

The same group in 2019 published a longitudinal study in which they collected dialysate from patients every 6 months for 2 years. Patients were divided into two groups according to PET results: stable (n = 7) or unstable (n = 4). EVs were isolated by size exclusion chromatography, and their protein content was analyzed by mass spectrometry combined with bioinformatics analysis. Their results demonstrated that changes in the protein content of vesicles occurred before PET could identify them, demonstrating the powerful potential of these vesicles as early biomarkers for detecting changes in PM function in these patients [65].

Pearson et al. demonstrated the importance of correct isolation of these EVs for protein analysis. In their study, isolation by differential centrifugation helped to identify more than 2000 proteins, usually masked by other abundant proteins in the dialysate. Furthermore, isolation by size exclusion chromatography further improved the identification of low-abundance proteins [92].

EVs can also be used as a marker of dialysis efficiency. Corciulo et al. analyzed the expression of the water channel Aquaporin 1 (AQP1) in mesothelial cells. In addition to confirming the expression, they demonstrated that AQP1 is released in the dialysate through exosomes where they found a strong positive correlation between the abundant presence of AQP1 in the dialysate and the fluid and solute transport parameters used to describe the efficiency of PD [66].

Akbari et al. demonstrated the presence of MVs derived from mesothelium in the effluent of patients on peritoneal dialysis for the first time. The mesothelial origin of these MVs was confirmed by Western blot, and they identified that the MV levels progressively increased during the 4 h dialysis cycle, suggesting that the exposure of the peritoneal membrane to the dialysis solution induces this process of MV formation [72]. The ability of these MVs to generate a response in the host remains a hypothesis to be tested.

EVs have extraordinary potential as biomarkers, especially in the context of peritoneal dialysis, where early identification of damage or failure of the peritoneal membrane can be crucial for continuing treatment. Collection of EVs from the dialysate is a painless procedure for the patient, and when combined with proteomic analysis could accurately indicate functional or metabolic alterations in the PM. Furthermore, they might be used as efficiency markers in peritoneal dialysis, therefore being useful tools in clinical practice. However, more studies are needed to confirm and expand their role in PD.

## 4. Isolation and Characterization of Extracellular Vesicles (EVs)

Isolation of EVs aims to separate EVs or a subtype of EVs (i.e., exosomes, MVs) from other non-vesicular components, enabling their specific study. The characterization is carried out to better understand the isolated EVs in order to elucidate the physicochemical properties and their composition (proteins, lipids, nucleic acids) according to what is intended to be studied. Several experimental approaches and methods have already been reported to achieve these goals. ISEV elaborated the Minimal Information for Studies of EVs 2018 (MISEV2018) which proposes the primary methods for isolating and characterizing EVs [93]. Here, we describe some methodological approaches to consider when endeavoring to isolate and characterize EVs, and the challenges that these approaches pose.

### 4.1. Isolating EVs

EVs are isolated from various types of medium such as in vitro cell culture media, tissues, and biological fluids [130]. For example, EVs released by mesothelial cells can be obtained from dialysate in peritoneal dialysis [57,65]. Notably, the isolation method used influences the yield and purity of the EVs or their subtype to be studied and this may affect downstream analysis [131,132]. Also, the separation of EVs can use more than one method, especially if greater specificity is required [93,133]. However, using two or more isolation methods can reduce the yield of EVs while decreasing contaminants such as soluble proteins and lipoproteins [131,134].

One of the most used methods for isolating EVs is differential ultracentrifugation (DUC), in which the solution containing the EVs is subjected to several centrifugation steps with increasing speed to pellet the EVs. The isolation of EVs by DUC usually consists of a step with low-speed centrifugation (1000 RCF) to remove cells and larger particles, followed by another intermediate speed spin (20,000 RCF) to collect larger EVs, and finally high-speed ultracentrifugation (100,000 RCF) to isolate smaller EVs [135]. However, it is worth noting that several parameters can affect the type, purity, and yield of EVs, such as rotor type, g-force, centrifugation time, sedimentation rotor angle, and viscosity of the sample [136,137,138,139]. Considering these parameters, possible adaptations to the centrifugation protocol, such as changing the centrifugation time, can improve the separation of the required EVs [137]. For instance, viscous samples need more time and greater ultracentrifugation speed [137].

Another method based on centrifuging samples is density gradient ultracentrifugation (DGUC). In this method, EVs are separated by a density gradient using sucrose, iohexol, or iodixanol under centrifugal force. The density gradient can be continuous or discontinuous (in layers), with a progressive increase in density from the top to the bottom of the tube in both [140]. Data from the literature indicate that the density of EVs varies between 1.1 and 1.19 g/mL [105,131,133,134].An improvement in yields and purity of EVs obtained with DGUC compared to ultracentrifugation was reported [141,142]. DGUC is also used to separate mitochondria and other cell organelles [143]. However, the disadvantages of this method are the long centrifugation time and sample loss.

EVs can be obtained by size exclusion chromatography (SEC), in which the solution (mobile phase) passes through a porous resin (stationary phase) in a column, and the EVs are separated based on size. In SEC, the smaller molecules enter the pores of the resin and their elution is delayed, while the larger molecules do not enter the pores and are eluted earlier [144]. This allows EVs and proteins to have distinct retention times, which reduces protein contamination of isolated EVs. Indeed, studies indicate that SEC applied to isolated exosomes from blood plasma contained low contamination, especially with albumin [145,146,147].

Ultrafiltration also separates EVs in which the sample is filtered through a nanopore membrane with molecular weight cut-off values, which can be separated by pressure, centrifugation, or vacuum [148,149]. The filtration allows the smaller particles to pass while the larger ones are retained in the membrane [149]. However, structural changes in the vesicles and membrane clogging may occur [136,150,151]. Ultrafiltration also can be used along with ultracentrifugation, particularly to separate EVs according to size [152].

### 4.2. Characterizing EVs

Characterizing EVs is essential to assess and relate possible biological effects and identify biomarkers, especially in pathological conditions. The characterization should use multiple methods considering the analysis objectives and the limitations of each approach to better understand the properties and composition of EVs [93].

#### 4.2.1. Electron Microscopy

Electron microscopy methods are frequently used to individually visualize the EVs and characterize their morphology and size. The ultrastructure of the EVs is observed using transmission electron microscopy (TEM) analyzers. This method consists of an electron beam which passes through an ultra-thin sample, and the scattered electrons are detected by the analyzers forming an image of the EVs. [153,154]. Compounds such as osmium tetroxide can be used to improve the contrast in TEM [155]. Another method is scanning electron microscopy (SEM), in which the surface topography of the EVs is analyzed also providing information about their chemical composition. In this method electron beams interact with the matter present on the surface of the sample, generating signals captured by a detector, in which secondary and backscatter electrons contribute to image formation [153,154]. However, it is interesting to point out that EVs may be altered by the processing steps of the samples for electron microscopy, which includes fixation, vacuum, and dehydration procedures [153]. Thus, cryo-electron microscopy (cryo-EM) evaluates samples which are vitrified at very low temperatures to minimize these interferences, without the need for chemical fixation or dehydration [153,156]. Cryo-EM also demonstrates the morphology and size of EVs, but has the advantage of preserving the hydrated native state of EVs [148,153,154].

#### 4.2.2. Nanoparticle Tracking Analysis

Nanoparticle tracking analysis (NTA) is widely used to describe the size distribution and concentration of EVs. In this method, a laser is applied under the sample, and the Brownian motion of the EVs makes it possible to detect its size and the concentration in the solution [157]. The NTA has a camera attached to track and record the displacement of each particle [157]. Nanoparticle tracking analysis (NTA) enables characterizing particles around 30 nm visualized by laser light scattering. The Stokes–Einstein equation can calculate the mean particle size based on Brownian motion. The equation evaluates that the size of a particle is in inverse proportion to its diffusion [158].

#### 4.2.3. Dynamic Light Scattering

Like NTA, dynamic light scattering (DLS) also evaluates the size distribution of EVs. DLS uses a laser which penetrates the sample, and the light scattering caused by the EVs in the solution due to the Brownian motion is captured by a detector, thereby ascertaining the hydrodynamic radius of the particles present in the sample [157]. However, unlike NTA, DLS measures bulk scattering [159,160].

#### 4.2.4. Flow Cytometry

Flow cytometry can also analyze EVs detected by light scattering and fluorescent labeling [152]. Even though it is a standardized and robust method for analyzing cells at a rate of 1000 cells per minute, flow cytometry in EVs is a great challenge. EVs scatter light at intensity 10 times less than polystyrene beads typically used for calibration due to their small size and low refractive index difference with the solution [161]. Thus, EVs smaller than 500 nm are detected in clusters, increasing the sensitivity above the defined detection limit, resulting in single-particle detections of EVs with sizes above 500 nm as well as warm detections, resulting in inaccurate measurements of results. Some solutions have been described to overcome this problem, such as promoting serial dilutions to achieve a linear correlation between the degree of dilution and the measured concentration [162] and the bead-based EVs assay. In this method, EVs are captured by the granules, immunostained, and subjected to analysis by flow cytometry [155,156]. Another method for analyzing EVs is utilizing an imaging flow cytometer, which uses flow cytometry and fluorescent labeling detected by charge-coupled device cameras with better performance than conventional flow cytometers [154,157,163]. In addition, high-sensitivity flow cytometers have been developed to analyze nanoscale particles [134,154,158,159,160].

#### 4.2.5. Proteomic, Transcriptome, Lipidomic and Metabolomic Approaches

Protein content is widely used in characterizing EVs as well as examining biomarkers. Cell-type specific proteins can be used to confirm the cellular origin of EVs. MISEV2018 recommends analyzing transmembrane or GPI-anchored proteins and cytosolic proteins to indicate the presence of lipid bilayer structures that encompass cytosolic material [93]. Another recommendation of MISEV2018 is to analyze the presence of contaminating proteins in the sample (i.e., albumin and apolipoproteins A1/2 and B), indicating the purity of the EVs [93]. Therefore, antibody-based techniques such as western blot, enzyme-linked immunosorbent assay (ELISA), and flow cytometry are often used to assess target-protein content [93]. These methods are based on the interaction of labeled antibodies with target proteins. The single EV analysis (SEA) method was developed to individually analyze protein biomarkers present in EVs. In this method, EVs are immobilized on a microfluidic chip, and then the target proteins are labeled with fluorescent antibodies, which enables labeling multiple targets at the same time [164].

Mass spectrometry techniques are extensively used in order to determine the proteomic profile of EVs. In summary, EVs are lysed and the proteins are subjected to enzymatic digestion with subsequent separation of the peptides in the mass spectrometer [159,165,166]. The proteomic study of EVs has an important role in the research of biomarkers in EVs that would normally be masked by abundant soluble proteins [65,167].

EVs also carry various RNAs such as mRNAs, miRNAs, and long RNAs molecules [168,169]. It is suggested that these RNAs have a regulatory effect on EVs recipient cells and may be potential biomarkers [135,159,170]. However, the number of RNA molecules per EV may be low, as previously reported [171,172,173]. RNA extraction from EVs can be performed using phenol-chloroform and column-based techniques [174,175]. The RNA content of EVs is obtained by RNA sequencing (RNA-seq) analyses [169,176]. In turn, quantitative reverse transcription-polymerase chain reaction (RT-PCR) and droplet digital PCR techniques (ddPCR) are often used to analyze specific RNA sequences [177,178,179,180].

The lipid content of EVs also plays a vital role as their composition differs under physiological and pathological conditions [151,181,182]. Importantly, EVs have bioactive lipids in their composition, such as ceramide, which may induce responses in recipient cells [183,184]. Thus, EVs lipidomics can contribute to understanding the biological mechanisms of EVs and identify potential biomarkers [151,185]. Mass spectrometry-based platforms and thin-layer chromatography (TLC) methods are used to study EVs lipidome profiling [186,187,188].

More recently, the metabolomic study of the content of EVs has gained ground [189]. Some studies have identified significant metabolites in EVs in pathological conditions, such as cancer [190,191]. However, it is necessary to use metabolite extraction protocols and define the analytical platform to study the metabolome of EVs using ultra-performance liquid chromatography-tandem mass spectrometry (UPLC-MS/MS) and gas chromatography-mass spectrometry (GC-MS) [163,190,192].

#### 4.2.6. Fourier Transform Infrared (FTIR) and Raman Spectroscopies as Diagnostic Tools

Given the potential to provide essential information on the composition and structural conformation of specific molecular species, vibrational spectroscopy techniques such as FTIR and Raman spectroscopies have become valuable tools in the past two decades [193]. Likewise, there is potential for their spectra to be translated into clinical research, establishing diagnoses and forensic techniques when combined with data analysis [194].

The basis of vibrational spectroscopy is defined by the interaction of radiation from a light source with the chemical bonds of a sample generating a unique signature designed into a spectrum, providing information for performing in qualitative and quantitative analyses of any compound [193]. The principal regions of biological interest in the infrared spectra are generally the amide I/II peaks and the fingerprint region at 900–1900 cm^−1^ [194]. Furthermore, according to Balan et al., the Raman technique presents special benefits including speed in data acquisition, being capable of providing information at the molecular level, being non-destructive and analyzing samples in aqueous solutions since water produces a weak Raman scattering, and it is important to study biological samples where researchers can investigate the ionization behavior, pH change, or amino acid shape. Thus, changes in normal biological systems reflect different vibrational spectral regions [193]. Consequently, within the field of biomedical analysis, the spectra obtained are tissue-specific spectroscopic signatures, characteristic of the histological state of the sample [195] or fluid composition [196]. However, biological samples are essentially complex since they are composed of different lipids, nucleic acids, proteins, and carbohydrates, where the spectra are the consequence of the absorption or diffusion characteristic of the bands of each of these. Therefore, vibrational spectroscopy techniques have become an important strategy in biomedical analysis, considering their experimental accessibility and for being a non-invasive method. Also, they require minimal sample preparation, high molecular sensitivity, speed and low cost [193].

FT-Raman spectroscopy has been explored in several diagnostic experiments [194], including cancer diagnosis (breast, cervical tumors, prostate, gastrointestinal and skin), neurological problems, diabetes, atherosclerosis, red blood cells infected with malaria, and monitoring osteoarthritis and rheumatoid arthritis in different experimental models, and also in renocardiac syndrome induced by renal ischemia and reperfusion [197]. These vibrational procedures are less invasive than traditional biopsies, have higher specificity, and are capable of detecting deviations in protein content at different stages of the disease progression [193].

Studies carried out by Nepomuceno et al. demonstrated that monitoring the Tyrosine and Tryptophan bands at 1558, 1616, and 1625 cm^−1^ is a viable and advantageous way to predict fatality in CRS both in vivo or in vitro using real-time multiplexing. Both amino acids are precursor molecules for the formation of uremic toxins such as PCS and IS, markers of kidney injury, whose increase is strongly correlated to cardiovascular mortality [197].

Recent studies have also shown that the IR spectroscopy–based protein quantification can be successfully adapted to experimental practice to analyze EVs. In contrast, vibrational spectroscopy presents a reagent-free alternative to traditional colorimetric protein determination assays and demands no special sample preparation to explore EVs [198]. According to results obtained by Paolini et al., FTIR also has the potential to promptly characterize EV subpopulations [199], suggesting it as an attractive complement or alternative method for understanding EVs in healthy and pathological situations.

## 5. Conclusions and Future Perspectives

The burden of kidney disease compromises the well being of around 10% of all adults in the world. Despite its high prevalence, we still have important gaps to fill in terms of the underlying mechanisms of the disease, diagnosis and treatment. EVs are a promising tool to improve the quality of care in kidney disease patients. EV have been consistently and successfully extracted from blood, urine, and more recently from peritoneal dialysate. We have a great opportunity with EV to identify new biomarkers capable of reducing CKD progression in the early stages of the disease, as well as the associated cardiovascular complications, and furthermore identify PD patients at greater risk of peritoneal membrane failure and the development of encapsulating peritoneal sclerosis. For this, more studies are needed focusing on the impact and opportunities of EVs in the CKD and CRS setting, and their contribution to heart–kidney interactions. The presence of kidney damage and the release of EVs are important factors that have been observed leading to dysfunction of several cell types, being closely related to inflammation, thrombosis, vasoconstriction, and atherosclerosis. The isolation of EVs from a patient’s body fluids is simple and painless, providing a promising tool for early diagnosis of these pathologies.

## Figures and Tables

**Table 1:** Classification of cardio-renal syndrome.

Denomination	Description
Type I	Heart failure causing acute kidney injury
Type II	Chronic heart failure causing chronic kidney disease
Type III	Acute kidney injury causing acute heart disease
Type IV	Chronic kidney disease causing chronic heart failure
Type V	Systemic condition causing heart and kidney disease

Adapted from Ronco et al., 2018 [10].

**Table 2:** Studies using extracellular vesicles in peritoneal dialysis.

Reference	*n*	EVs Type	Methodology
[72]	8	Microvesicles	Electron microscopy, nanoparticle tracking analysis (NTA), flow cytometry, procoagulant activity, and Western blot.
[92]	13	Multiple extracellular vesicles	Isolation by differential centrifugation and size exclusion chromatography. Vesicle analysis by electron microscopy, NTA, dynamic lighting scattering (DLS), and tandem mass spectrometry.
[57,65]	11	Multiple extracellular vesicles	Extracellular vesicles were isolated by size exclusion chromatography, and proteomics was analyzed by mass spectrometry (LC-MS/MS)
[66]	30	Exosomes containing Aquaporin 1 (AQP1)	Exosomes were isolated by differential centrifugation and identified by Western Blot. Expression of AQP1 in mesothelial cells was done by immunofluorescence. The quantification of AQP1 in the dialysate was performed using a commercial enzyme-linked immunosorbent assay (ELISA) kit.
